# GeneSPIDER2: large scale GRN simulation and benchmarking with perturbed single-cell data

**DOI:** 10.1093/nargab/lqae121

**Published:** 2024-09-18

**Authors:** Mateusz Garbulowski, Thomas Hillerton, Daniel Morgan, Deniz Seçilmiş, Lisbet Sonnhammer, Andreas Tjärnberg, Torbjörn E M Nordling, Erik L L Sonnhammer

**Affiliations:** Department of Biochemistry and Biophysics, Stockholm University, Science for Life Laboratory, Box 1031, Solna 171 21, Sweden; Department of Immunology, Genetics and Pathology, Uppsala University, Uppsala 751 85, Sweden; Department of Biochemistry and Biophysics, Stockholm University, Science for Life Laboratory, Box 1031, Solna 171 21, Sweden; Department of Biochemistry and Biophysics, Stockholm University, Science for Life Laboratory, Box 1031, Solna 171 21, Sweden; Department of Biochemistry and Biophysics, Stockholm University, Science for Life Laboratory, Box 1031, Solna 171 21, Sweden; Department of Cell and Molecular Biology, Karolinska Institutet, Solna 171 77, Sweden; Department of Biochemistry and Biophysics, Stockholm University, Science for Life Laboratory, Box 1031, Solna 171 21, Sweden; Department of Neuro-Science, University of Wisconsin-Madison, Waisman Center, WI 53705, USA; Department of Mechanical Engineering, National Cheng Kung University, No. 1 University Road, Tainan City 701, Taiwan; Department of Biochemistry and Biophysics, Stockholm University, Science for Life Laboratory, Box 1031, Solna 171 21, Sweden

## Abstract

Single-cell data is increasingly used for gene regulatory network (GRN) inference, and benchmarks for this have been developed based on simulated data. However, existing single-cell simulators cannot model the effects of gene perturbations. A further challenge lies in generating large-scale GRNs that often struggle with computational and stability issues. We present GeneSPIDER2, an update of the GeneSPIDER MATLAB toolbox for GRN benchmarking, inference, and analysis. Several software modules have improved capabilities and performance, and new functionalities have been added. A major improvement is the ability to generate large GRNs with biologically realistic topological properties in terms of scale-free degree distribution and modularity. Another major addition is a simulation of single-cell data, which is becoming increasingly popular as input for GRN inference. Specifically, we introduced the unique feature to generate single-cell data based on genetic perturbations. Finally, the simulated single-cell data was compared to real single-cell Perturb-seq data from two cell lines, showing that the synthetic and real data exhibit similar properties.

## Graphical Abstract

**Graphical Abstract:**
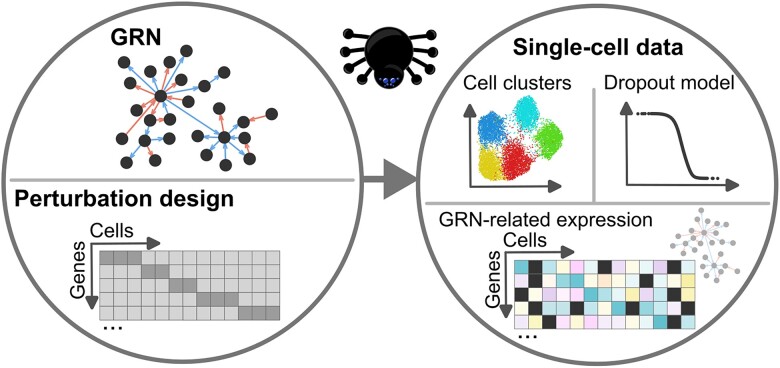

## Introduction

Recent advances in single-cell transcriptomics have opened the field of inferring gene regulatory networks (GRNs) from this data type (1–4). GRNs have for instance been applied to explore the single-cell cancer regulome of transcription factors (5) or miRNAs (6). They have also been used to investigate transcriptional regulation in the immune cell response to infection with SARS-CoV-2, which led to insights into disease mechanisms (7,8). Nevertheless, to evaluate computational tools for GRN inference, simulations of ground truth GRN and data are essential (1,9). In the past years, various single-cell data simulators have been developed (10–12). However, perturbations have not been considered in these implementations.

**Figure 1.**
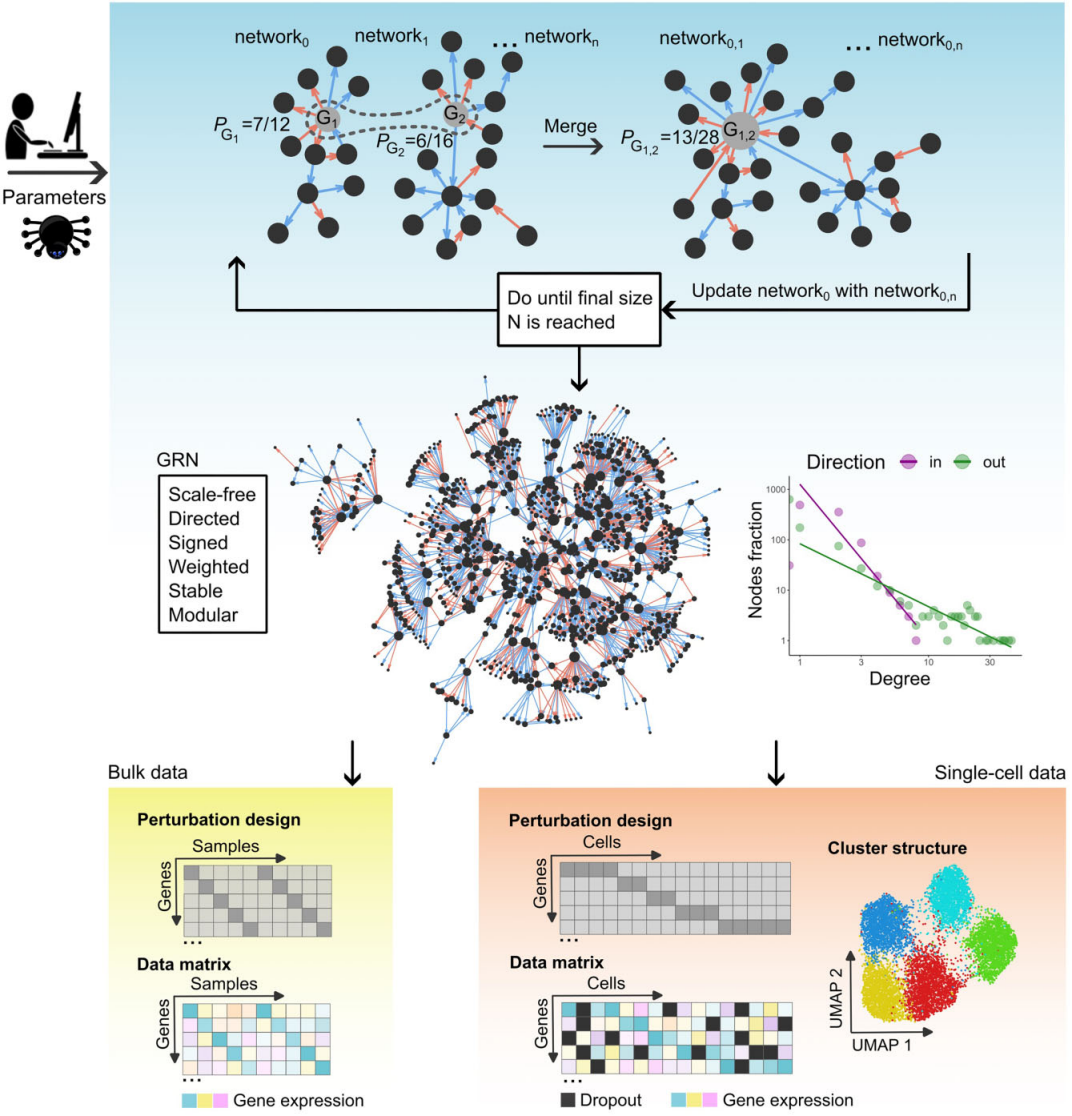
Generation of stable large scale-free GRNs and biologically realistic data with GeneSPIDER2. Some parameters are depicted in the figure including *P_G_*, the degree-based attachment probability of a node $G$, and $N$, the total number of nodes (or genes) in the final GRN.

In this work, we present GeneSPIDER version 2 (GS2), a freely available MATLAB toolbox. The first version of GeneSPIDER (13) was mainly aimed at generating small GRNs, typically below 100 genes. Scaling up to thousands of genes led to issues with speed, topology, and stability. To resolve these problems, a new algorithm was designed that adopts small GRNs which are stitched together via selected nodes into a large and stable scale-free GRN (Figure 1). Another major new capability is generating perturbed single-cell-like data with noise specific to single-cell RNA-seq (scRNA-seq). To evaluate the properties of the simulated single-cell data, we compared it to real single-cell expressions measured with the Perturb-seq protocol for K562 (14) and Calu-3 (15). To our knowledge, GS2 is the only tool that allows for simulations of knockdown perturbations in single-cell data using a GRN as the basis for data generation. The extended functionality makes GS2 well-suited for challenging tasks in benchmarking GRN inference methods with perturbed single-cell data for small and large scale-free GRNs.

## Materials and methods

### Generating large scale-free GRNs

Large GRNs are constructed by stitching together small scale-free GRNs, referred to as subGRNs, that can be generated quickly and robustly. The stitching procedure relies on the Barabasi–Albert model (16) such that nodes between two subGRNs become common with a probability based on their degree (Figure 1). We also improved the algorithm for generating small scale-free GRN topologies by changing it to the Barabasi–Albert model and allowing for individual control of out- and in-degree distributions such that topologies from known GRNs can be mimicked (Supplementary Figure S1 and Supplementary Figure S2). The probability $P$ of stitching to a node with a given degree $x$ is defined as $P = c{{x}^\alpha }$, where $c$ is a constant and $\alpha$ is the exponent of the power law distribution, which can be tuned in GS2. In addition, the distribution of link signs is based on the TRRUST database version 2 (17) including 62% positive interactions, i.e. activations, are drawn with a probability of 0.62.

### Simulating single-cell data

GS2 uses the same principle as the first version of GeneSPIDER for simulating knockdown perturbations in bulk gene expression data (13). For this the noise-free gene expression matrix $X$*∈ R^|e|×|g|^* is calculated as $X = - {{A}^{ - 1}}P$ where $A$*∈ R^|g|×|g|^* is an adjacency matrix of a GRN, with eigenvalues with negative real part, and $P$*∈ R^|e|×|g|^* is the matrix of the experimental (or perturbation) design, i.e. defining which gene is perturbed in which experiment for $e$ experiments/cells and $g$ genes (18). The values of $X$ can be interpreted as noise-free logarithmic fold-changes of bulk gene expression relative to the control. To model technical noise when simulating the data, random Gaussian noise ${{E}_G}$, drawn based on the signal-to-noise ratio (SNR), is added to noise-free fold-change data (${{X}_{FC}}$) as follows: $Y = {{X}_{FC}} + {{E}_G}$. Several variants of SNR are available in GS2 (see Supplementary Material).

In GS2, we use simulated log fold-changes from Y to model single-cell data by converting them to raw counts and modelling a dropout noise. To achieve that, we create a so-called simulated control count (SCC) matrix (${{M}_{SCC}}$) that is constructed in several phases. In the first phase, the mean of each row in ${{M}_{SCC}}$ (${{\mu }_{NB}}$), i.e. the SCC of a single gene, is defined as a value drawn from a negative binomial distribution (19) with a user-defined probability ${{p}_{NB}}$ and the number of successes $R$ set to 1. To obtain a non-discrete mean, a value between 0 and 1 is drawn from a uniform distribution and added to ${{\mu }_{NB}}$. In the second phase, the distribution of counts for each gene in ${{M}_{SCC}}$ is drawn from a lognormal distribution (20) using ${{\mu }_{NB}}$ and the standard deviation of $Y$. To obtain a clustered data structure, the ${{M}_{SCC}}$ values are drawn from a lognormal distribution according to the cell's membership in a cluster (Supplementary Figure S3). For each cluster, two expression means are calculated: ${{\mu }_1}$ that is cluster-specific, and ${{\mu }_2}$ outside the cluster, where the relationship is that ${{\mu }_1} >{{\mu }_2}$. Then the expression of cells within clusters is adjusted based on ${{\mu }_1}$ and ${{\mu }_2}$. This allows data to keep different average expressions for clusters so they are distinguishable in further analysis. The strength of the distance between clusters can be tuned by the user.

In the next step, the variance of each gene in $Y$ ($\sigma _Y^2$) is calculated, and rows in ${{M}_{SCC}}$ are sorted according to $\sigma _Y^2$ such that the gene with the highest $\sigma _Y^2$ is paired with the highest ${{\mu }_{NB}}$ (21). The rationale for this step is that genes with a high mean are known to exhibit a high variance. To mimic the raw counts of unique molecular identifiers (UMIs) (22), the inverse logarithm (default base 10) of the values in $Y$ are multiplied by ${{M}_{SCC}}$ as follows ${{Y}_{UMI}} = Y \odot {{M}_{SCC}}$. The resulting ${{Y}_{UMI}}$ can be considered as a synthetic perturbation-based and cluster-specific matrix of discrete counts.

In the final step, to model single-cell-specific noise, we impute zeros on ${{Y}_{UMI}}$ relying on the dropout model by (21). The binary dropout matrix ${{E}_D} = [ {{{e}_{ij}}} ]$*∈ R^|e|×|g|^* is estimated based on the dropout probability:


(1) \begin{eqnarray*}P\left( {k = 0} \right) = {{\left( {\frac{{{{\phi }^{ - 1}}}}{{\mu + {{\phi }^{ - 1}}}}} \right)}^{{{\phi }^{ - 1}}}}\end{eqnarray*}

where $\phi$ is a dispersion parameter that controls the variance and $\mu$ is the average expression of the gene. ${{E}_D}$ is constructed by drawing $q$ from a uniform distribution and setting


(2) \begin{eqnarray*}{{e}_{ij}} = \left\{ {\begin{array}{@{}*{1}{c}@{}} 0\\ 1 \end{array}} \right.\ \begin{array}{@{}*{1}{c}@{}} {{\mathrm{if\ }}P\left( {k = 0} \right) >q}\\ {{\mathrm{otherwise}}} \end{array}\end{eqnarray*}

To create synthetic single-cell data with dropouts (${{Y}_{SC}}$), UMI counts are inflated with zeros as follows:${\mathrm{\ }}{{Y}_{SC}} = {{Y}_{UMI}} \odot {{E}_D}$. Optionally, ${{Y}_{SC}}$ can be converted back to a logarithmic fold-change. Details about noise models in GS2 can be found in the Supplementary Text.

## Results

GeneSPIDER includes a range of GRN inference applications that can exploit the experimental perturbation design used to generate the gene expression data. Perturbation-based GRN inference methods (Supplementary Table S1) have been shown to outperform other approaches (23). As described above, major improvements to GS2 include the simulation of large realistic scale-free GRNs and scRNA-seq data. Moreover, we also listed minor improvements to GS2 and included a comparison with other single-cell data simulators (see Supplementary Text, and Supplementary Tables S1 and S2). To investigate the scale-freeness of GRNs (24), we analysed the degree distributions of in- and out-going links. In contrast to the first version of GS (Supplementary Figure S4), GS2 can generate large scale-free GRNs (Supplementary Figure S5) with distinct in and out degree distributions that are similar to various biological GRNs (Supplementary Figure S1 and Supplementary Table S3). Moreover, the modularity of GS2 GRNs can now be tuned with alpha and subGRN size (Supplementary Figure S6B) to make them similar to biological GRNs (Supplementary Figure S6A), which was not possible with the previous version (Supplementary Figure S6C).

To evaluate the construction speed of large scale-free GRNs we measured the running time of constructing GRNs with varying sparsity, i.e. average node degree, and compared it to the former GeneSPIDER version. The results show that GS2 can generate a stable GRN of 20 000 genes in 10–20 min, while the previous version was only able to generate a GRN of 1000 genes at this time (Supplementary Figure S7).

**Figure 2.**
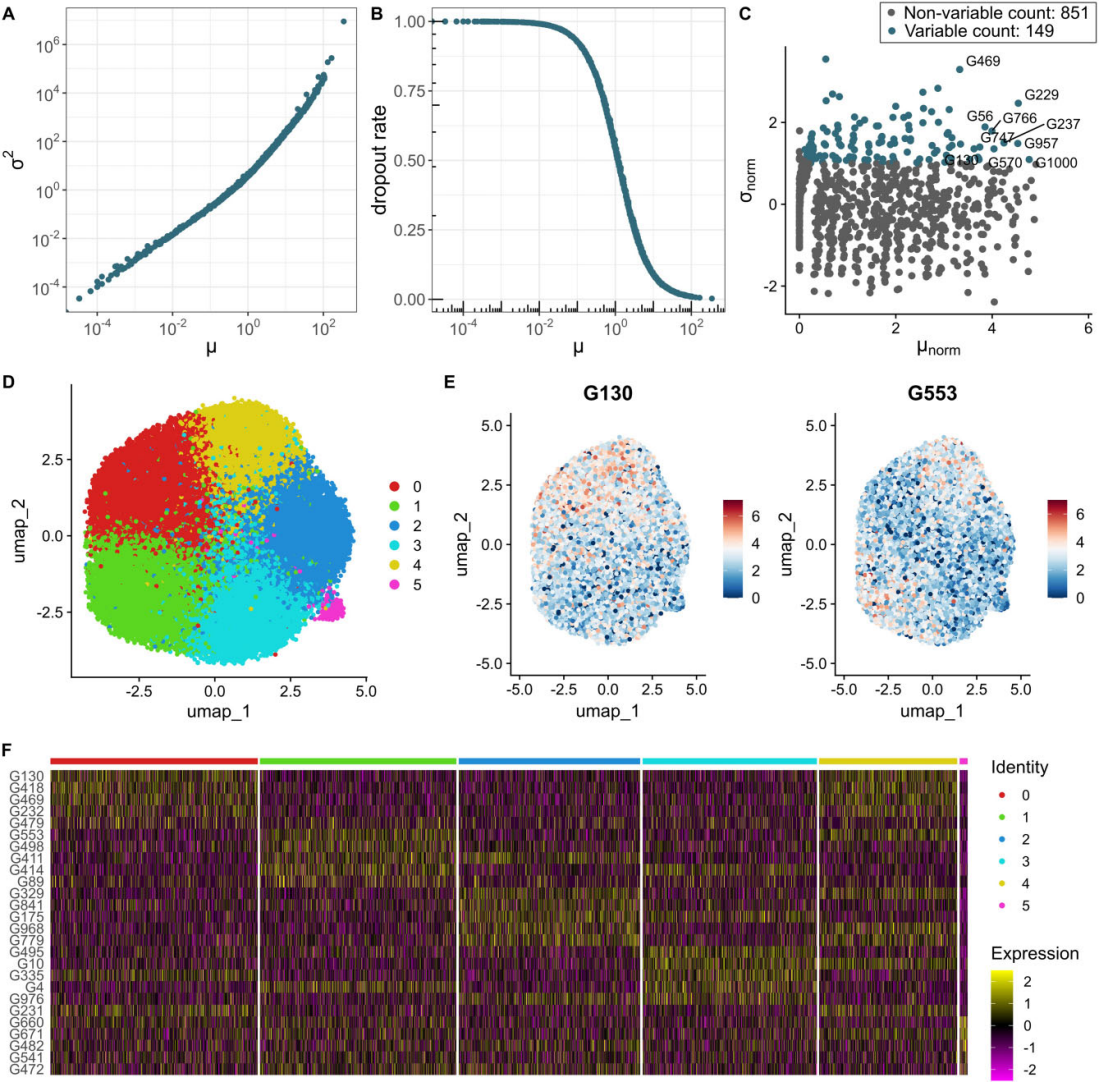
Data summary of GS2 synthetic single-cell data with default parameters where SNR_vov is 0.1 and 50 000 cells were simulated for 1000 genes. (**A**) Relationship between variance (${{\sigma }^2}$) and mean ($\mu$) expression. (**B**) Relationship between dropout rate and mean expression. (**C**) Standard deviation (${{\sigma }_{norm}}$) versus mean (${{\mu }_{norm}}$) expression plot produced by Seurat 5.0.1 on normalized data for the 5% most variable genes. (**D**) Uniform Manifold Approximation and Projection (UMAP) for dimension reduction for the number of clusters set to 5. (**E**) Cluster-specific expression of two example genes. (**F**) Gene expression patterns across clusters for genes with the highest variability. (C–F) Subplots were constructed with the Seurat 5.0.1 package.

**Figure 3.**
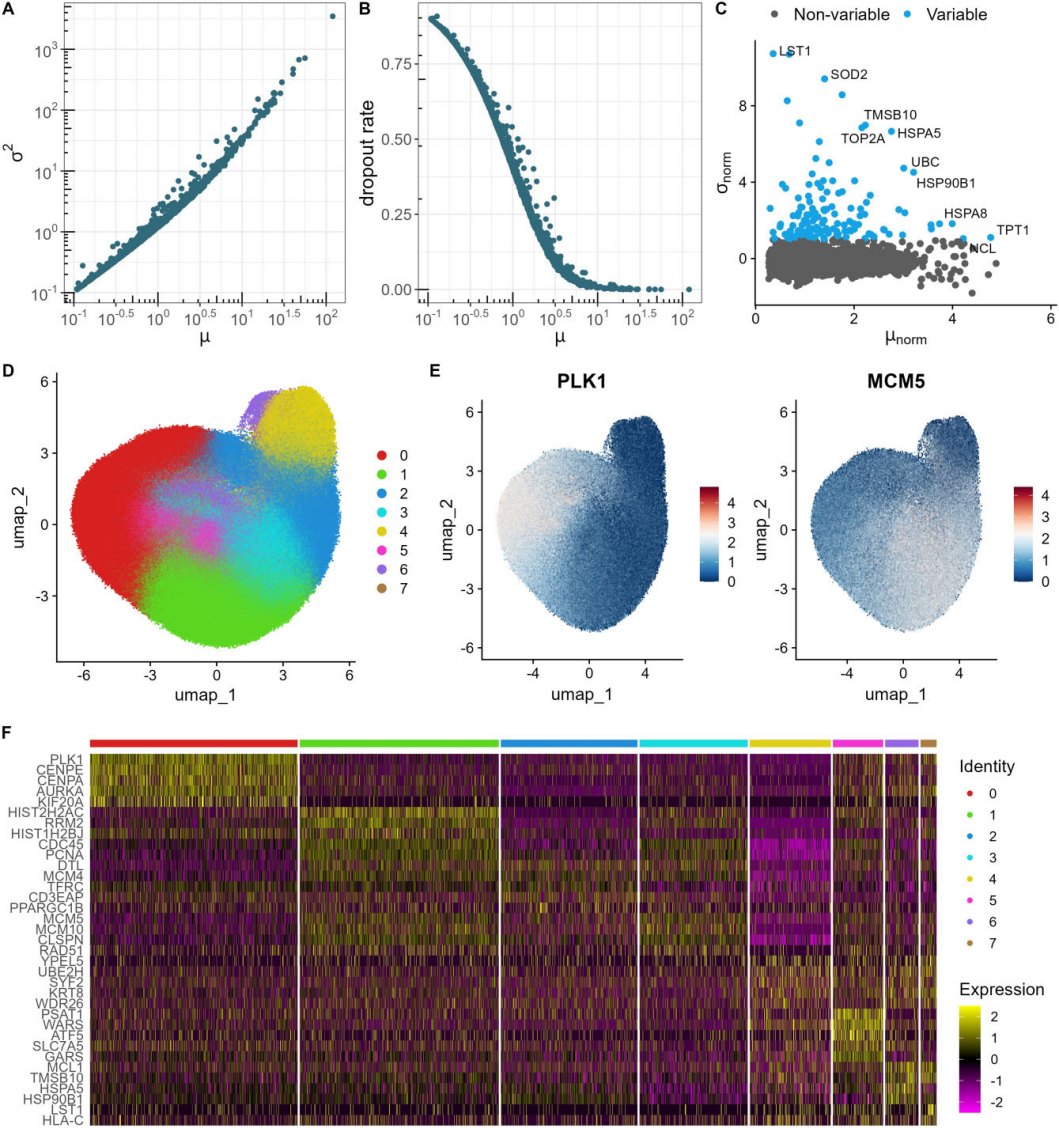
Data summary of CRISPRi Perturb-seq scRNA-seq data from K562 cells. (**A**) Relationship between variance (${{\sigma }^2}$) and mean ($\mu$) expression. (**B**) Relationship between dropout rate and mean expression. Note that genes with high dropout rates were removed during preprocessing by the authors of this data. (**C**) Standard deviation (${{\sigma }_{norm}}$) versus mean (${{\mu }_{norm}}$) expression plot produced by Seurat 5.0.1 on normalised data for the 5% most variable genes. (**D**) Uniform Manifold Approximation and Projection (UMAP) for dimension reduction for the number of clusters set to 5. (**E**) Cluster-specific expression of two example genes. (**F**) Gene expression patterns across clusters for genes with the highest variability. (C–F) subplots were constructed with the Seurat 5.0.1 package.

To assess the quality and properties of perturbed single-cell data simulated by GS2, we compared it to CRISPRi Perturb-seq scRNA-seq data from Calu-3 (15) and K562 (essential set) (14), in which 183 and 1868 genes were knocked down, respectively. In addition, we evaluated CRISPR CROP-seq scRNA-seq data for HCC38 and HCC1143 cell lines where 50 genes were knocked out (25). In this assessment, we used SNR_vov (the variance of signal over the variance of noise) for simulations as it exhibited more variability for larger data. The comparison suggests that salient properties of real single-cell data are well reflected in GS2 synthetic data (Figures 2–3 and Supplementary Figures S8–S10). In all data sets, the mean and variance relationship match the negative binomial distribution (Figure 2–3A and Supplementary Figure S8-S10A). Furthermore, dropout probabilities depend on mean expression (Figures 2–3B and Supplementary Figures S8–S10B), and the normalised mean versus standard deviation relationships show similar distributions (Figures 2–3C and Supplementary Figures S8–S10C). In addition, the synthetic data has a clustered structure (Figure 2D, F) that is observed in real data sets as well (Figure 3D, F and Supplementary Figures S8–S10D and Supplementary Figure S8–S10F). It results in a set of cluster-specific genes that are usually overexpressed in a specific cluster (Figures 2–3E and 2–3F and Supplementary Figures S8–S10E and Supplementary Figures S8–S10F).

Next, we analysed the perturbation effect in the synthetic single-cell and real scRNA-seq (Supplementary Figures S11 and S12) data. In general, distributions display similarity across all data sets, although real single-cell data had a weaker perturbation effect than synthetic single-cell data. A possible explanation is that real single-cell data is more affected by noise (26) and biological variability (27). This may have led to a decreased number of cells that were correctly perturbed in experimental data. Moreover, we showed that in GS2, the user is able to control the perturbation strength (Supplementary Figure S12A, B). By changing the perturbation strength in the perturbation design matrix, higher P-values and closer E-distances (28) to unperturbed cells were obtained (see Supplementary Text). For the experimental scRNA-seq knockdown data, we found that the E-distances differed strongly between the Calu-3 and K562 cells, in accordance with their different perturbation strengths (Supplementary Figure S12C, D).

Finally, as GS2 contains various operations, including randomization techniques and conversions between fold-change and raw counts, we measured the Pearson correlation between input (*Y*) and output (*Y_SC_*) fold-change values (Supplementary Figure S13). This examination showed a very high correlation, about 1, which ensures a strong connection between the single-cell data and the GRN used to generate it.

## Discussion

GS2 is an open source MATLAB toolbox that has been equipped with additional modules and functions to meet new challenges in the GRN field. The scope of transcriptomics simulations was extended to produce perturbed single-cell data simulations, which is a unique feature among all single-cell data simulators.

We showed that GS2 simulates scRNA-seq data similar to techniques such as CRISPRi Perturb-seq or CRISPR Crop-seq. However, it could be retooled in the future to simulate other perturbed omics data types, such as Perturb-ATAC (29). In such a case, the perturbation design matrix should be adjusted to gene regions instead of genes. Afterward, such data could be converted to gene expression allowing the tool to create matched and perturbed multi-omics data sets.

While investigating experimental data, we can observe clusters that are close to each other (Figures 2D–3D and Supplementary Figures S8D–S10D). As cells of single types were evaluated, we expect to see close clusters, for example, K562 data was created from lymphoblast cells (30). This property is also reflected in synthetic data. Here, such clusters may appear due to various pathways that are activated via knock down/out or because of differences in the strength of perturbations. We could observe this behaviour also in the synthetic data. Specifically, in the simulations, we frequently obtained additional clusters that came out from the perturbation effect itself (cluster 5 in Figure 2D).

The perturbation strength varies across data sets. In the example synthetic data set, we assumed that all knockdowns were performed successfully and cells were affected by noise-derived variation (Figure 2). In experimental data sets, the perturbation strength in cells was often weak or none, however a set of cells was perturbed successfully, i.e. near a peak around 0 (Supplementary Figure S11D and G). For example, K562 includes more successful perturbations than Calu-3 (Supplementary Figure S11D and G). This is also reflected in the E-test (28) where K562 includes more significantly perturbed cells than Calu-3 (Supplementary Figure S12C). It suggests that K562 is less noisy than Calu-3 (Supplementary Figures S11 and S12). We also showed that perturbation strength similar to K562 or Calu-3 can be achieved for simulated single-cell data. By comparing P value distributions from the E-test of simulated and experimental data, Calu-3 would correspond to about 50% unsuccessfully perturbed cells while K562 to about 0–25% (Supplementary Figure S12). Thus, to mimic the experimental data where cells lack of successful perturbation effect, we encourage users to design the perturbation matrix appropriately.

In GS2, we assumed that running one simulation to create single-cell data corresponds to a single batch. To obtain several batches, we recommend running the simulation several times with various noise levels. This should reflect a technical variation between batches. Importantly, this can be another feature of GS2 that may allow for the benchmark of batch effect correction tools and its influence on GRN inference.

As there are large differences between perturbed bulk RNA-seq and scRNA-seq data, it is crucial to design simulations appropriately. Therefore, properties such as noise model, perturbation design, and perturbation strength were adapted appropriately. Recently, large-scale single-cell perturbation-based gene expression datasets are starting to become available, for example, Perturb-seq of 9866 genes (14). It is therefore imperative to perform GRN and data simulation at a large scale with realistic properties and low computation times, which is now possible with GS2.

## Supplementary Material

lqae121_Supplemental_File

## Data Availability

GeneSPIDER2 runs under the MATLAB computing platform and is available under GPLv3 license at https://doi.org/10.5281/zenodo.10949060. Software source code and post-processed fold-change gene expression data with perturbation design matrices are publicly available at https://bitbucket.org/sonnhammergrni/genespider/. Raw single-cell RNA-seq data were uploaded by its authors on Figshare at https://doi.org/10.25452/figshare.plus.20029387.v1 and GEO (GSE208240 and GSE241115).
