# Supplementary material for: GeneSPIDER2: large scale GRN simulation and benchmarking with perturbed single-cell data
Source: NAR Genom Bioinform. 2024 Sep 18;6(3):lqae121. doi: 10.1093/nargab/lqae121 (PMC11409065; doi:10.1093/nargab/lqae121)
Supplement: lqae121_Supplemental_File [file lqae121_supplemental_file.pdf]

Supplementary material

GeneSPIDER2: large scale GRN simulation and benchmarking with perturbed single-cell data

Supplementary text 3
Preprocessing of real perturbed data sets 3
Minor updates in GeneSPIDER 3
Noise models 4
E-test estimation 4
Comparison to other GRN-based single-cell data simulators 5
Supplementary figures 6
Supplementary tables 19
Supplementary references 23

Supplementary text

Preprocessing of real perturbed data sets

With GeneSPIDER2 (GS2) we provide three examples of real gene expression datasets, one from bulk and two from single-cell perturbation experiments. The bulk data from cell line K562 in which 232 genes were knocked down with shRNA was retrieved from the ENCODE repository (1) with biosample IDs ENCBS405CUM and ENCBS131LFM for the first and second replicates, respectively. The ENCODE dataset was converted to a logarithmic fold-change. The first single-cell dataset from the Calu-3 cell line in which 183 SARS-CoV-2 host factors were knocked down by CRISPRi using the Perturb-seq protocol, and then infected with the SARS-CoV-2 virus, was obtained from GEO with accession number GSE208240 (2). The second single-cell dataset from the K562 cell line in which 2057 essential genes were knocked down by Perturb-seq was obtained from https://gwps.wi.mit.edu/ (3). Both single-cell data sets were preprocessed using standard procedures with the Seurat 5.0.1 R package (4), where filtration (min.cells = 3, min.features = 200) and normalization with *LogNormalize* option was performed (**Supplementary Figures S12**). Moreover, ribosomal, mitochondrial, hemoglobin and *MALAT1* genes were excluded. Next, only cells treated with single gene perturbations were selected. Then fold-changes were calculated as the ratio between the perturbed expression and the average expression in unperturbed control cells. Besides, for example data sets attached to the GS2 toolbox we corrected dropouts in two steps: 1) by imputing the mean expression from all cells with the same gene knocked out, and 2) for the remaining zeros by imputing the mean expression across all cells. This allowed us to create dropout-free datasets ready for inference.

Minor updates in GeneSPIDER

Except for the major changes described in the main text, many minor improvements were made in GS2. For instance, as the package Glmnet is no longer supported, we replaced it with internal MATLAB functions for LASSO, Elastic net, and Ridge regression. We further integrated the bootstrap-based FDR estimation method NestBoot (5) into GS2 and extended it with new inference methods as well as optimized and parallelized its code. Several new inference methods were added to GS2 so that the tool now contains 30 GRN inference methods (**Supplementary Table S1**). Given the popularity of the GENIE3 inference method (6), we added several decision tree-based algorithms, including CART, bagging, adaptive boosting, gentle adaptive boosting, and adaptive logistic regression. In addition, several other MATLAB functions were wrapped for perturbation-based inference, such as support vector machines, neural networks and various regression methods.

To increase the user-friendliness of GeneSPIDER, we built a tutorial website (https://sonnhammer-tutorials.bitbucket.io/genespider.html), which provides detailed explanations of all steps of GRN analysis, including GRN and data generation, GRN inference for synthetic and real data, and benchmarking of inference methods. Moreover, we integrated all third-party inference methods into GeneSPIDER2. Finally, GeneSPIDER includes real example knockdown datasets from the K562 cell line as bulk (1) and single-cell data (3), and from the Calu-3 cell line for single-cell data (2).

Noise models

In the previous version of GS, the main additive Gaussian-based noise model was defined as $SNR\_L = \frac{\Sigma_{min}(X_{FC})}{\sqrt{\chi^{-2}(\alpha, N \times M)\sigma^2}}$ where $\Sigma_{min}(X_{FC})$ is the smallest singular value of X_{FC}, $\chi^{-2}(\alpha, N \times M)$ is the inverse χ^2 distribution at level α with $N \times M$ degrees of freedom (number of genes × number of experiments), and σ^2 is the variance of the noise matrix (E_G) (7). In GS2, we implemented several new additive Gaussian models: 1) SNR_mosd is defined as $\frac{\mu_{signal}}{\sigma_{noise}}$ where μ_{signal} is the mean of absolute X_{FC} and σ_{noise} the standard deviation of the E_G, 2) SNR_mosd2 is defined as $\frac{\mu^2_{signal}}{\sigma^2_{noise}}$ where μ^2_{signal} is the squared mean of absolute X_{FC} and σ^2_{noise} the variance of the E_G, 3) SNR_vov that is defined as $\frac{\sigma^2_{signal}}{\sigma^2_{noise}}$ where σ^2_{signal} is the variance of X_{FC} and σ^2_{noise} the variance of the E_G (8) and 4) SNR_cov that is defined as $\frac{cov_{signal}}{\sigma^2_{noise}}$ where cov_{signal} is the mean covariance of X_{FC} and σ^2_{noise} the variance of the E_G. Based on these SNR methods, we calculate the standard deviation of noise (σ_{noise}) that is used to draw E_G.

E-test estimation

To show how the strength of perturbations can be controlled in simulations and how it looks in experimental single-cell data, we adapted the E-distance measure and performed an E-test as proposed by (9). However, we slightly modified the procedure by balancing the number of perturbed and unperturbed cells across iterations. This was done by drawing the same number of unperturbed cells as the number of perturbed cells that went through the knockdown of a given gene. In case the perturbed set of cells had more cells than the unperturbed set, a full unperturbed set of cells was taken. In E-test, for synthetic and Calu-3, we used 1000 repetitions, while for K562 100 repetitions due to the larger data size. In this test, we assumed that various fractions of perturbations were not performed successfully. In other words, 0%, 25%, 50%, 75%, and 100% of diagonal values in the perturbation matrix were assigned to a uniformly drawn random number between -0.5 and 0.5. This means a cell was simulated with low perturbation, none, or low increase of expression. The rest of the perturbations were set to -1, meaning that a cell was simulated with knockdown (Supplementary Figure S12). For example, 50% means that half of the diagonal elements in the perturbation design matrix are set to -1 and the remaining half are set to a number drawn between -0.5 and 05. In simulations, the unperturbed set of cells was additionally created for a given data and GRN with a perturbation design matrix diagonal values set all to a number drawn between -0.5 and 05. This allowed simulating a full set of unperturbed cells. Here, the E-distance was computed using the energy R package (10).

Comparison to other GRN-based single-cell data simulators

We compared GS2 against other simulation tools such as GRouNdGAN (11), SERGIO (12) and BoolODE (13) (**Supplementary Table S2**). In this comparison, GeneNetWeaver (14) was excluded as it is no longer supported. Importantly, note that GS2 is focused on gene knockdown, while other methods were designed for gene knockout. By comparing all tools, GS2 is the only one that simulates a scale-free network internally and allows controlling the size of the network and its modularity. The remaining tools rely either on ground truth GRNs, where subnetworks are selected upon some assumptions or use external tools for synthetic network generation. Furthermore, we observe that SERGIO and GRouNdGAN rely on reference single-cell datasets, i.e. examples of real gene expression data, which limits parameter settings such as noise level or clustering properties. As GS2 does not use reference data to simulate counts, users may freely control parameters such as SNR, number of clusters, and count distribution. Substantially, GS2 synthetic data are not biased by external factors that can be inherited from real single-cell reference data such as batch effects or other unknown variations. Among all investigated tools, only GS2 and BoolODE include a set of GRN inference methods. However, GS2 contains a much larger set of GRN inference methods that are developed to use the perturbation design.

Supplementary figures

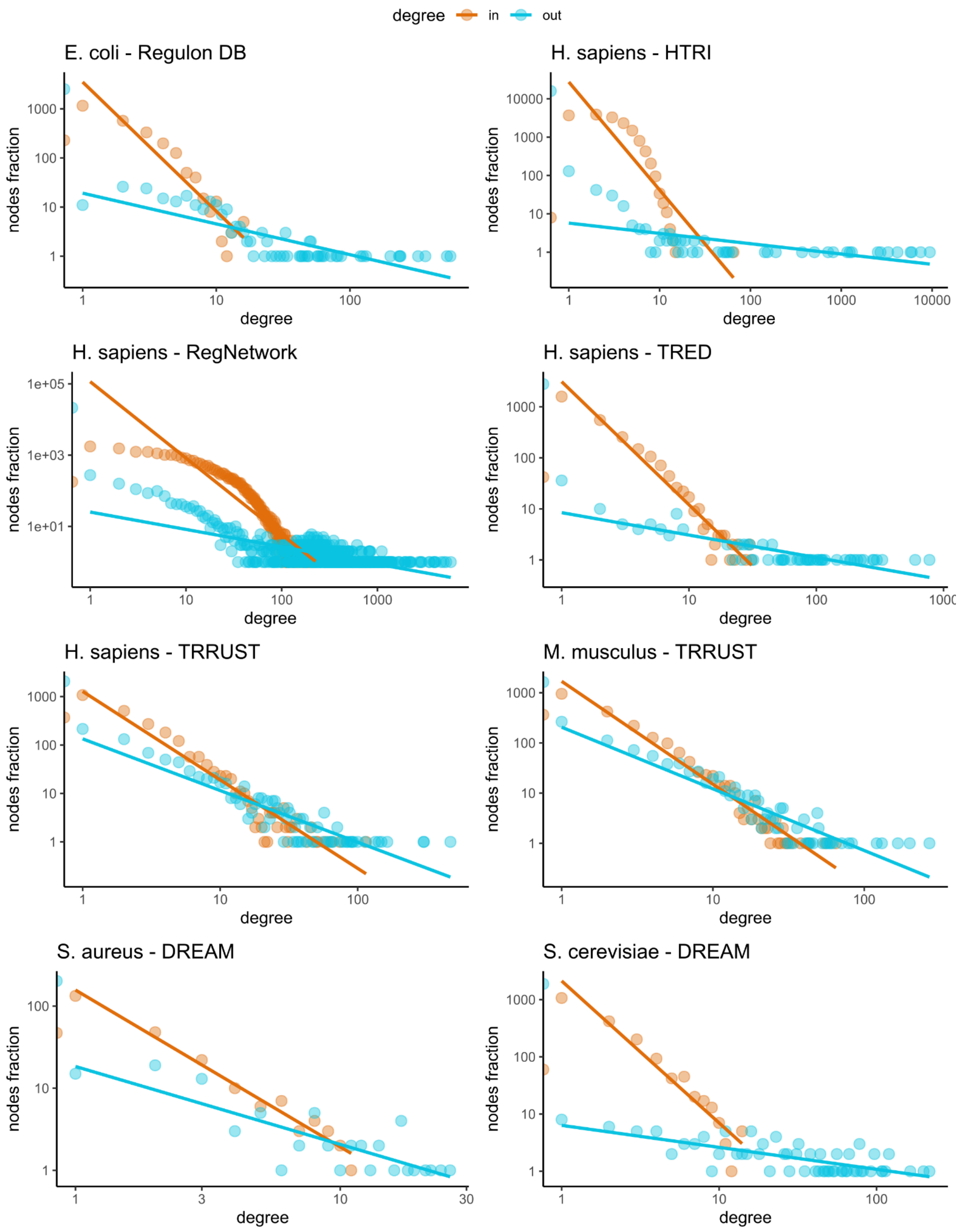

Figure S1. The distributions of in and out node degrees across eight gold standard networks for five organisms from RegulonDB, HTRI, RegNetwork, TRED, TRRUST and DREAM5 (for source database details see **Supplementary Table S3**). A linear regression line is shown for each degree distribution.

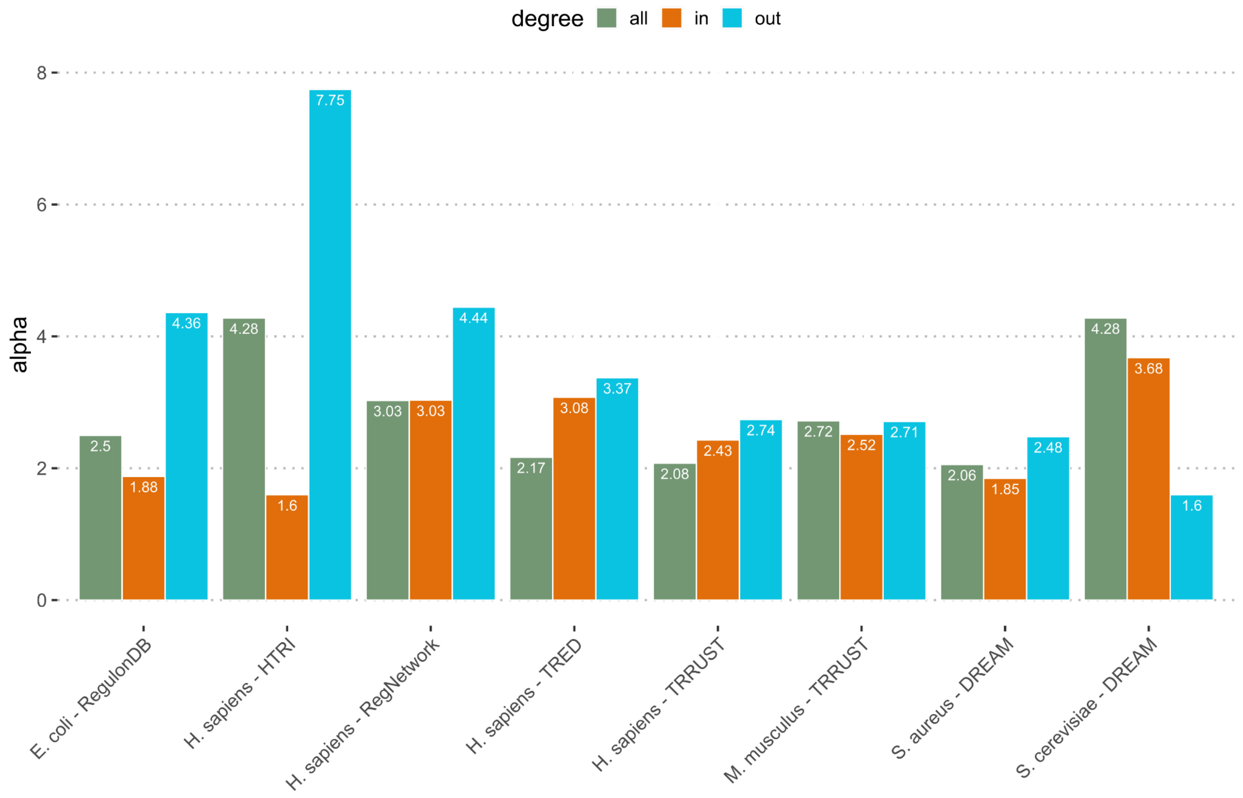

Figure S2. Power law α (y-axis) parameters estimated for all, in and out degrees across five various organisms from RegulonDB, HTRI, RegNetwork, TRED, TRRUST and DREAM5 (for source database details see **Supplementary Table S3**). The α parameters were estimated using the maximum likelihood approach with the 'plfit' option of *fit_power_law* function from the *igraph* 1.4.1 package (15).

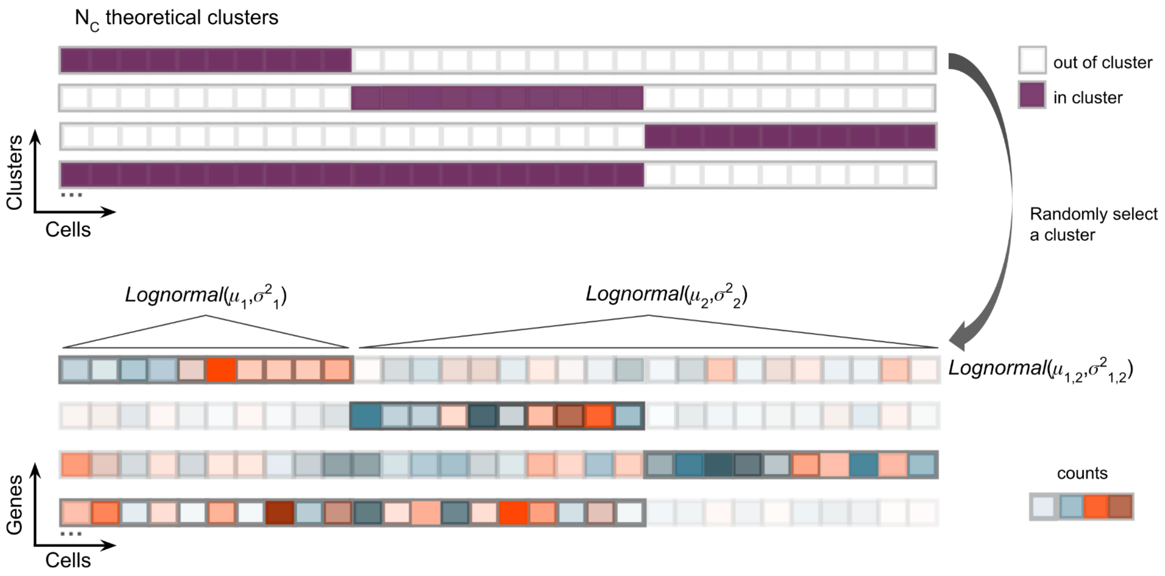

Figure S3. Model of creating single-cell control counts with cluster-specific averages and standard deviations. The theoretical clusters are built as binary vectors for all N_C combinations of clusters estimated as $\frac{(n_c+1)\times n_c}{2}$ where n_c is the user-defined number of clusters. Random selection of a cluster that is assigned to a given gene is based on a uniform distribution. A theoretical cluster manifests itself as cells with a mean expression (μ_1) higher than outside of the cluster (μ_2). Variation is marked as σ^2.

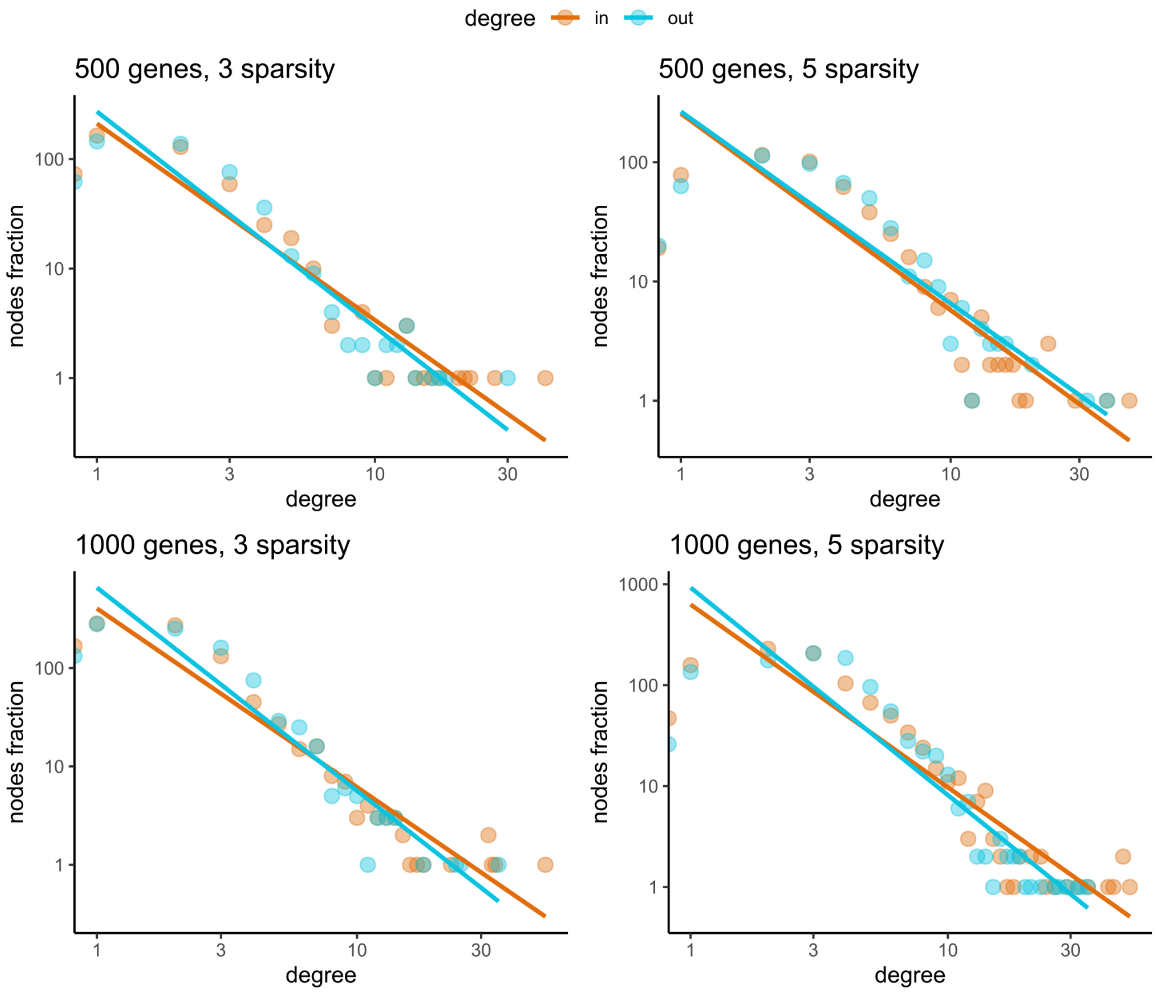

Figure S4. The distributions of in and out node degrees for large stable networks simulated with the first version of GeneSPIDER, for two different GRN sizes (500 and 1000 genes), and two different sparsities 3 and 5 links per node. A linear regression line is shown for each degree distribution.

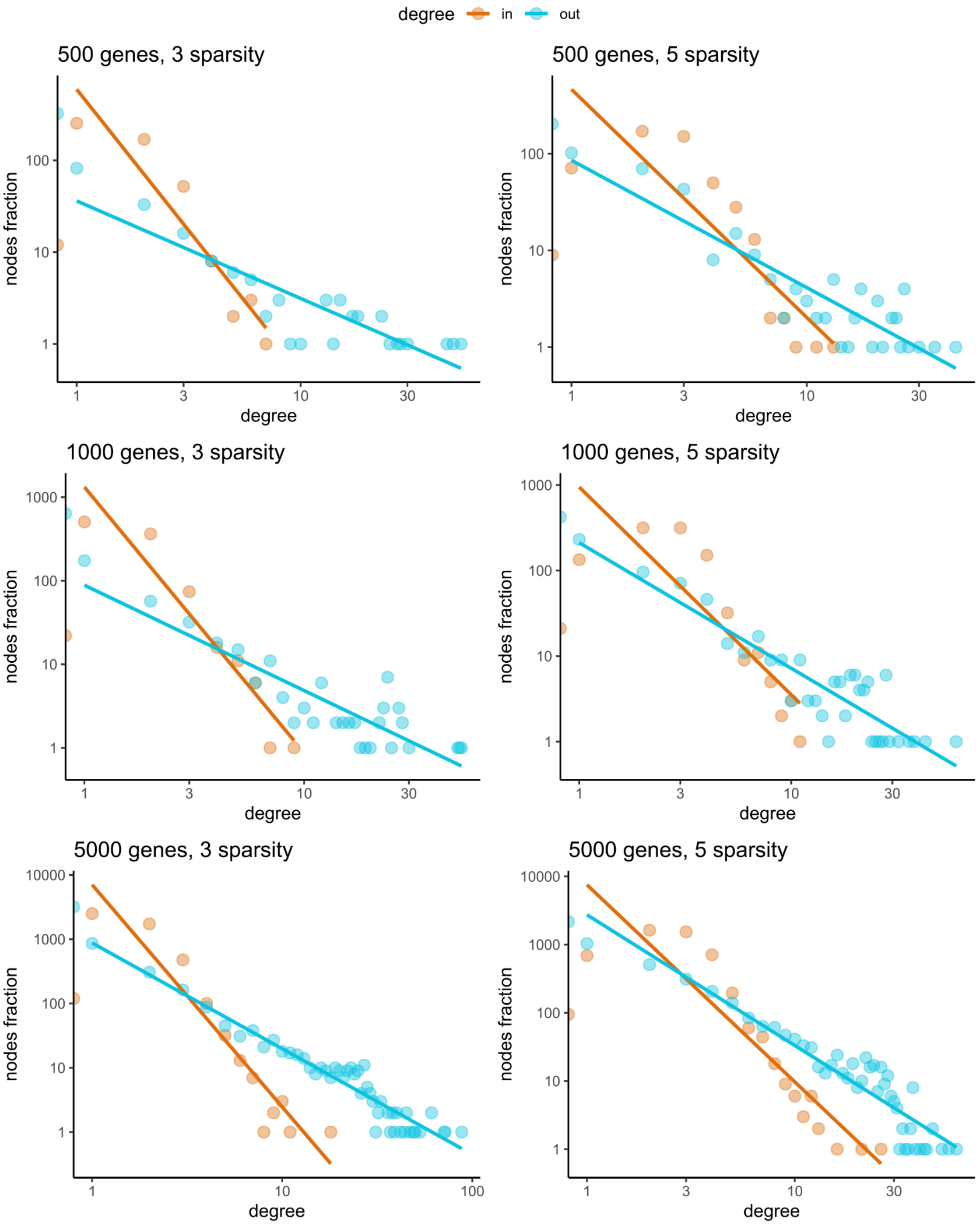

Figure S5. The distributions of in and out degrees for large stable networks simulated with GeneSPIDER2, for three different GRN sizes (500, 1000 and 5000 genes), and two different sparsities 3 and 5 links per node. A linear regression line is shown for each degree distribution.

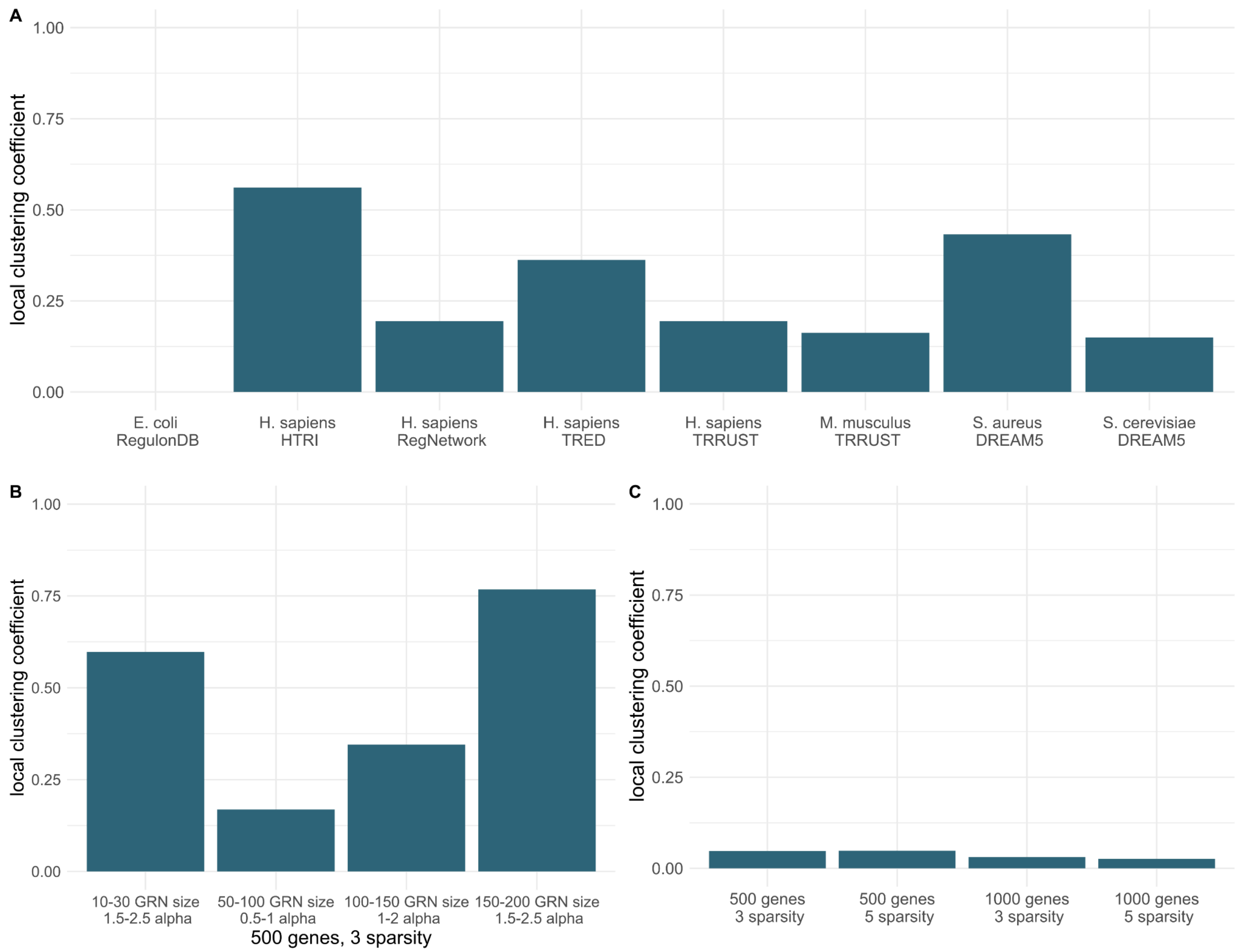

Figure S6. Comparison of modularity in terms of average local clustering coefficient for **A)** gold standard biological GRNs (for database details see **Table S2**) **B)** four example settings in GeneSPIDER2 GRNs and **C)** four examples from former GeneSPIDER GRNs. Local clustering coefficients were estimated using the transitivity function as implemented in the *igraph* 1.4.1 R package. Alpha (α) is the exponent of the power law distribution, and GRN size refers to the minimum and maximum number of genes in the subGRNs used to construct a 500-gene GRN.

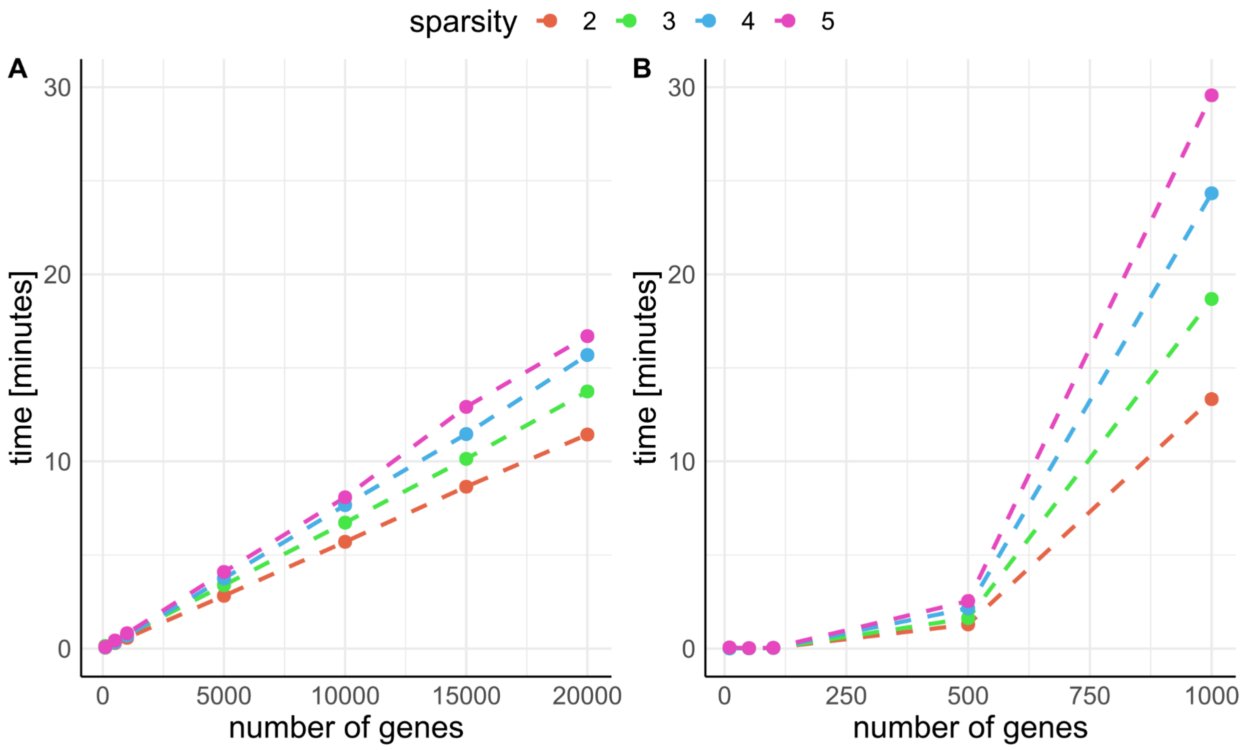

Figure S7. Comparison of real running time for networks of different sizes and at various sparsity, i.e. average degree for undirected GRN between **A**) GeneSPIDER2 and **B**) former version of GeneSPIDER for generating stable scale-free networks. The time was measured on a machine with 16 Intel Xeon E5620 2.40 GHz cores and 70 GB RAM.

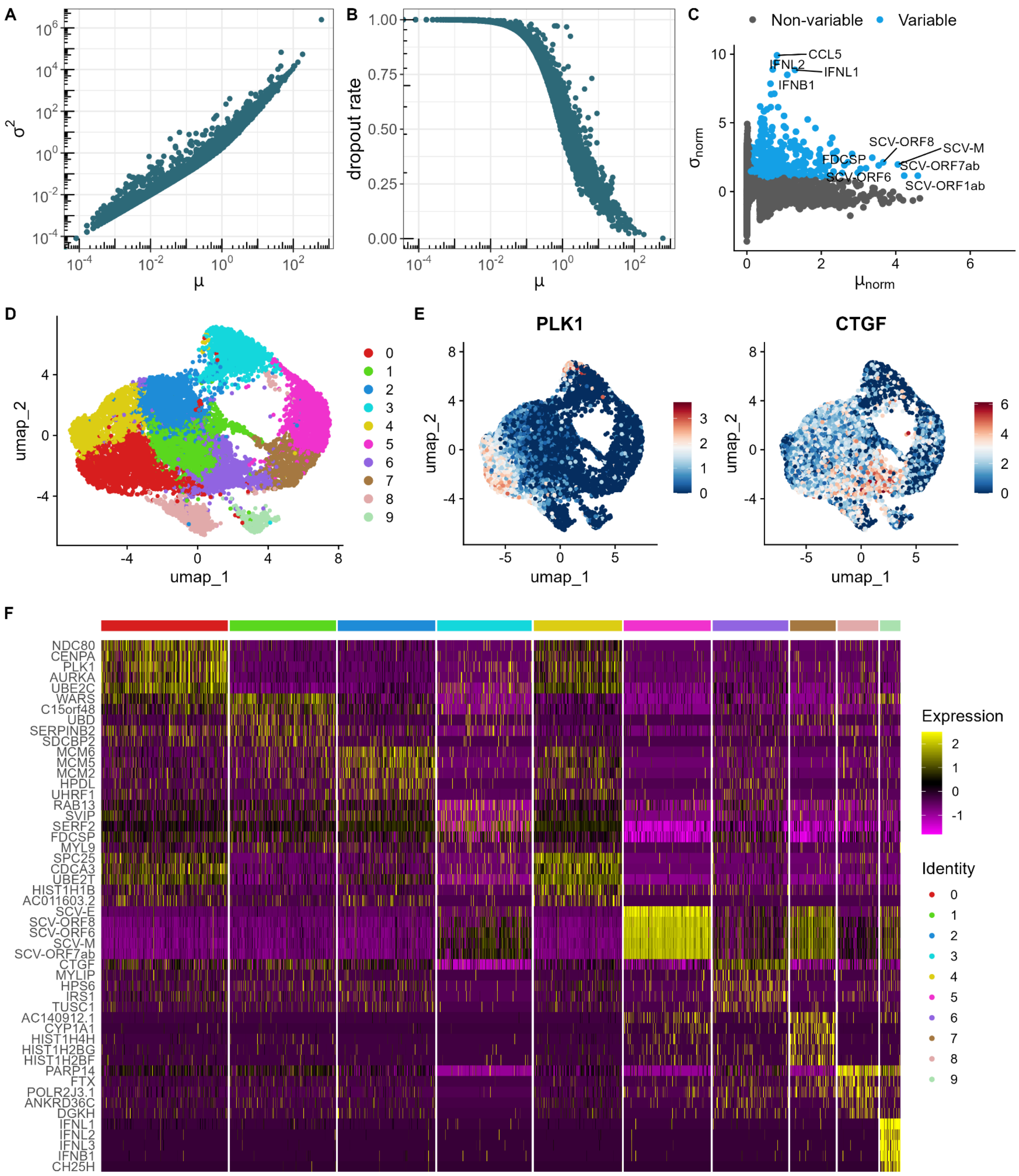

Figure S8. Data summary of CRISPRi Perturb-seq knockdown scRNA-seq data from SARS-CoV-2 infected Calu-3 cells (2). **A**) Relationship between variance (σ^2) and mean (μ) expression. **B**) Relationship between dropout rate and mean expression. **C**) Standard deviation (σ_{norm}) versus mean (μ_{norm}) expression plot produced by Seurat 5.0.1 on normalized data for the 5% most variable genes. **D**) Uniform Manifold Approximation and Projection (UMAP) for dimension reduction for the number of clusters set to 5. **E**) Cluster-specific expression of two example genes. **F**) Gene expression patterns across clusters for genes with highest variability. **C-F** subplots were constructed with the Seurat 5.0.1 package.

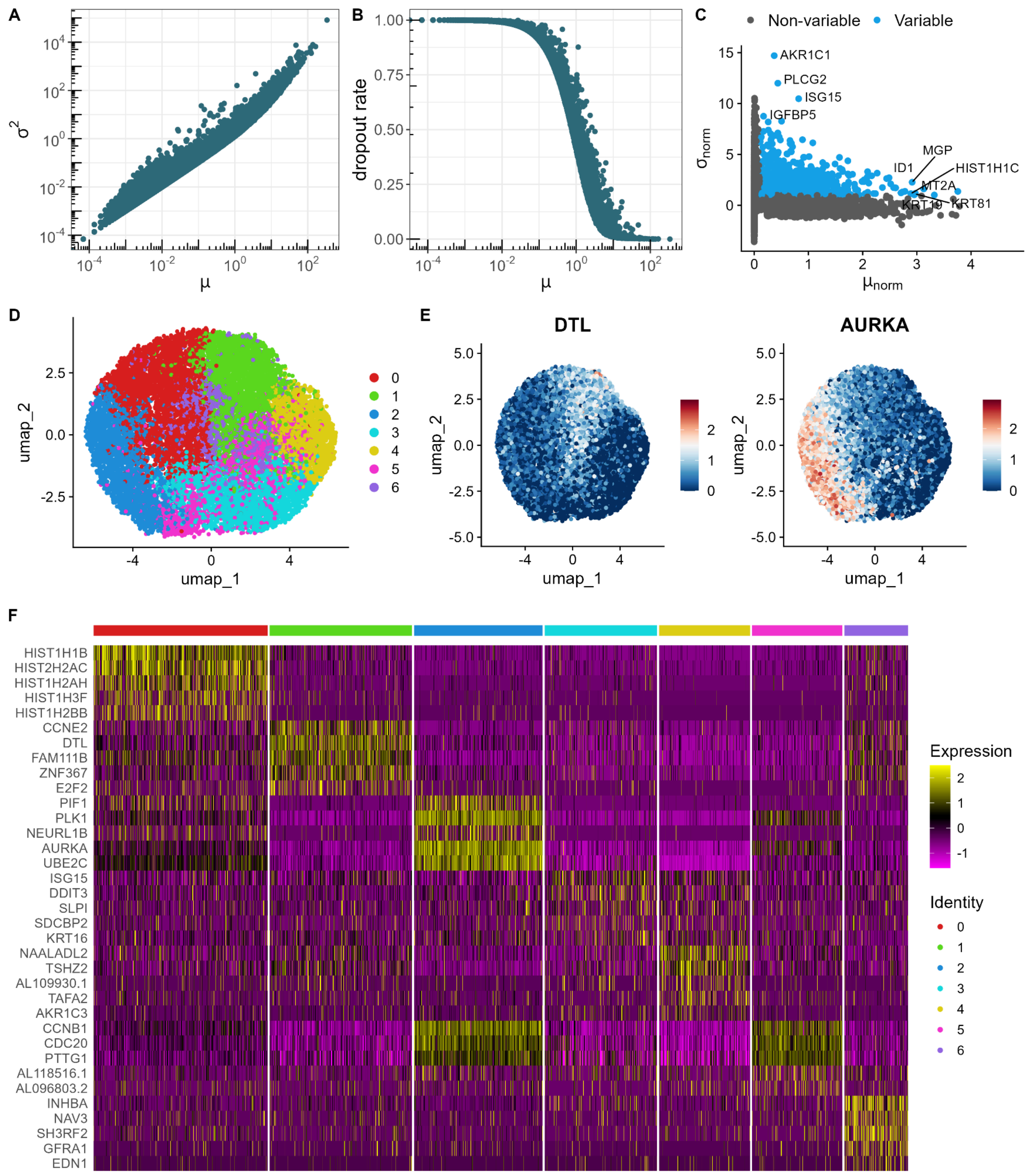

Figure S9. Data summary of CROP-seq knockout scRNA-seq data from HCC38 cells (2). **A**) Relationship between variance (σ^2) and mean (μ) expression. **B**) Relationship between dropout rate and mean expression. **C**) Standard deviation (σ_{norm}) versus mean (μ_{norm}) expression plot produced by Seurat 5.0.1 on normalized data for the 5% most variable genes. **D**) Uniform Manifold Approximation and Projection (UMAP) for dimension reduction for the number of clusters set to 5. **E**) Cluster-specific expression of two example genes. **F**) Gene expression patterns across clusters for genes with highest variability. **C-F** subplots were constructed with the Seurat 5.0.1 package.

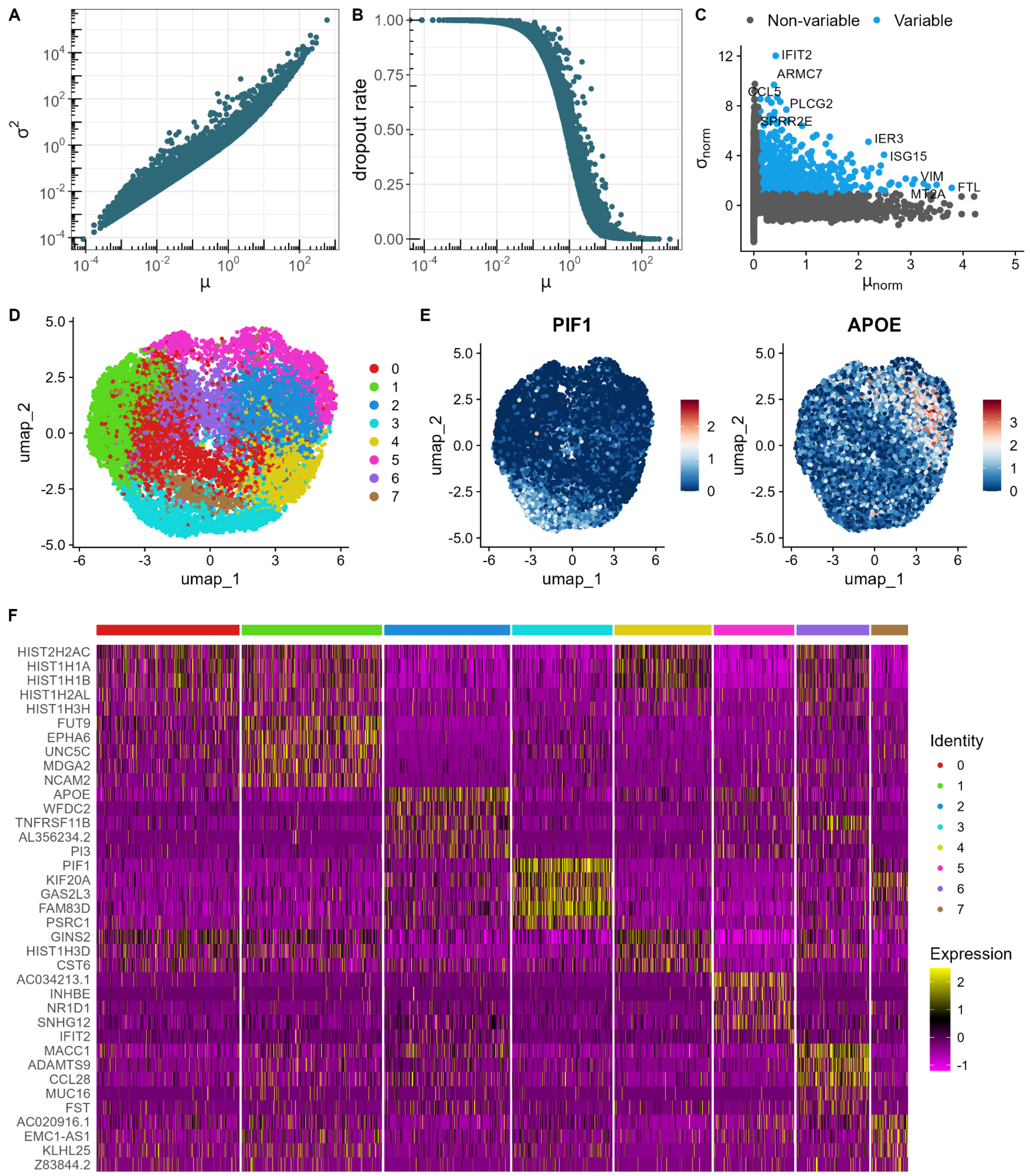

Figure S10. Data summary of CROP-seq knockout scRNA-seq data from HCC1143 cells (2). **A**) Relationship between variance (σ^2) and mean (μ) expression. **B**) Relationship between dropout rate and mean expression. **C**) Standard deviation (σ_{norm}) versus mean (μ_{norm}) expression plot produced by Seurat 5.0.1 on normalized data for the 5% most variable genes. **D**) Uniform Manifold Approximation and Projection (UMAP) for dimension reduction for the number of clusters set to 5. **E**) Cluster-specific expression of two example genes. **F**) Gene expression patterns across clusters for genes with highest variability. **C-F** subplots were constructed with the Seurat 5.0.1 package.

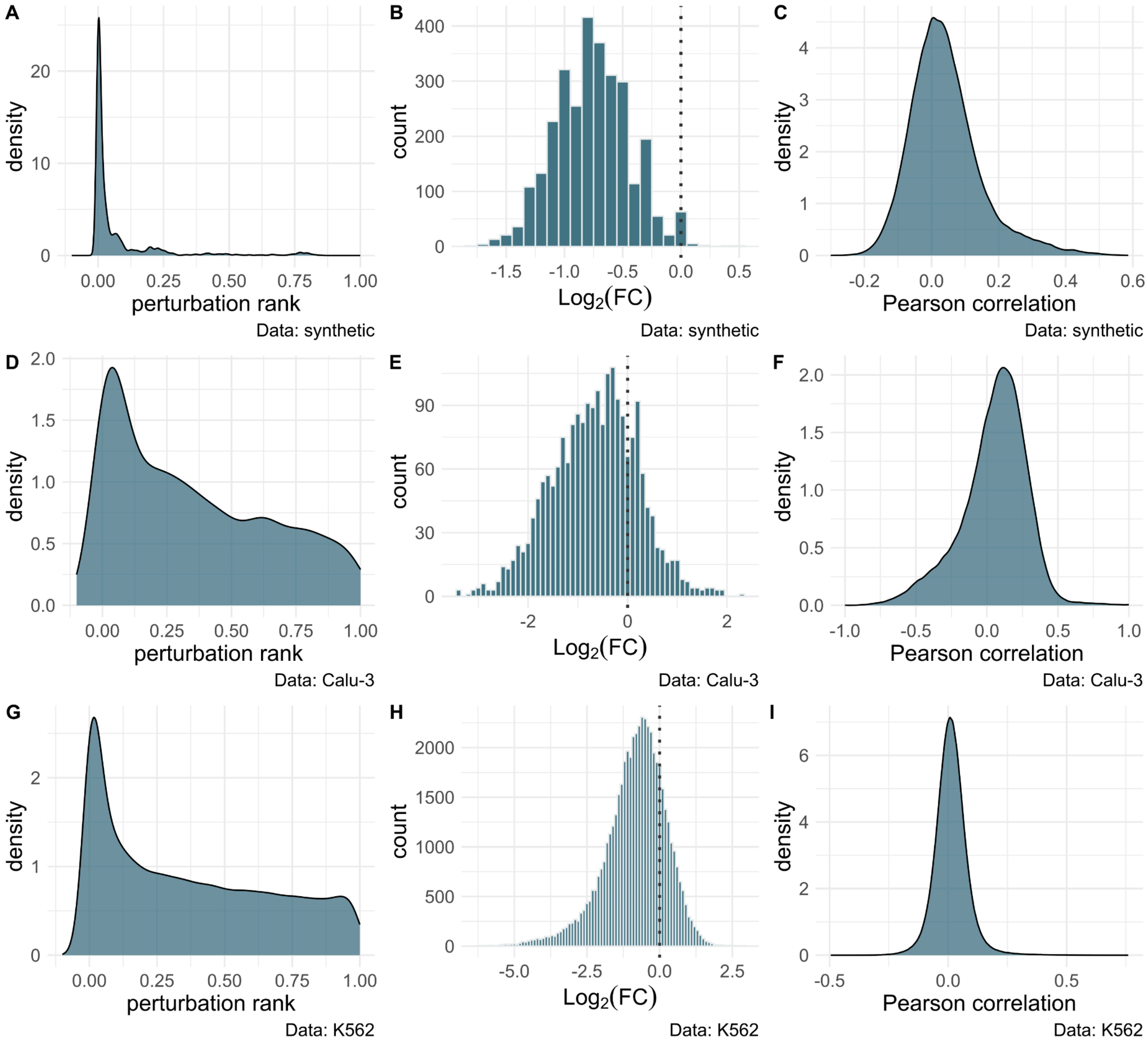

Figure S11. Overview of perturbation effect and replicates correlation for single-cell synthetic knockdown data from GS2 with 500 genes and 10000 cells for SNR_vov 0.05, Calu-3, and K562. **A, D, G**) Perturbation rank of the target genes, calculated as the percentage of genes that have a stronger knockdown effect. **B, E, H**) Log_2 fold-change expression of the target genes. **C, F, I**) Pearson correlation between replicates, i.e. cells where the same gene was perturbed.

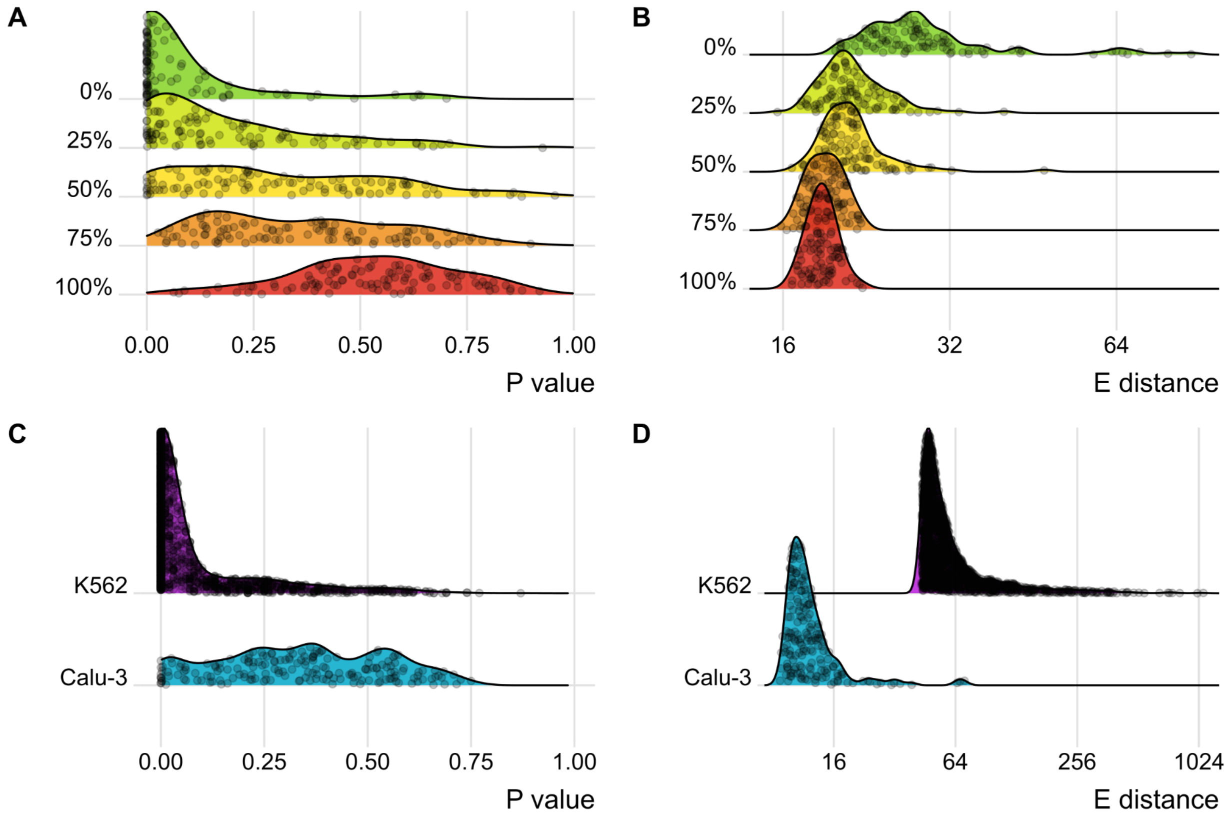

Figure S12. Overview of the perturbation effect strength control in synthetic data **A, B)**, and in real knockdown data **C, D)**. Percentages in **A** and **B** correspond to the fraction of diagonal elements in the perturbation design matrix that were set to random numbers between -0.5 and 0.5 while the remaining elements were set to -1. P values were calculated based on the E-test of being different from unperturbed cells. The E-distances to unperturbed cells in **B** and **D** are displayed on a $\log_2$ scale. The synthetic data sets in **A** and **B** were generated for 100 genes where the knockdown of each gene was repeated 50 times. The GRN with an average sparsity of 3 links/node was used. The SNR_vov level was set to 0.05 and the rest of the parameters were left as default.

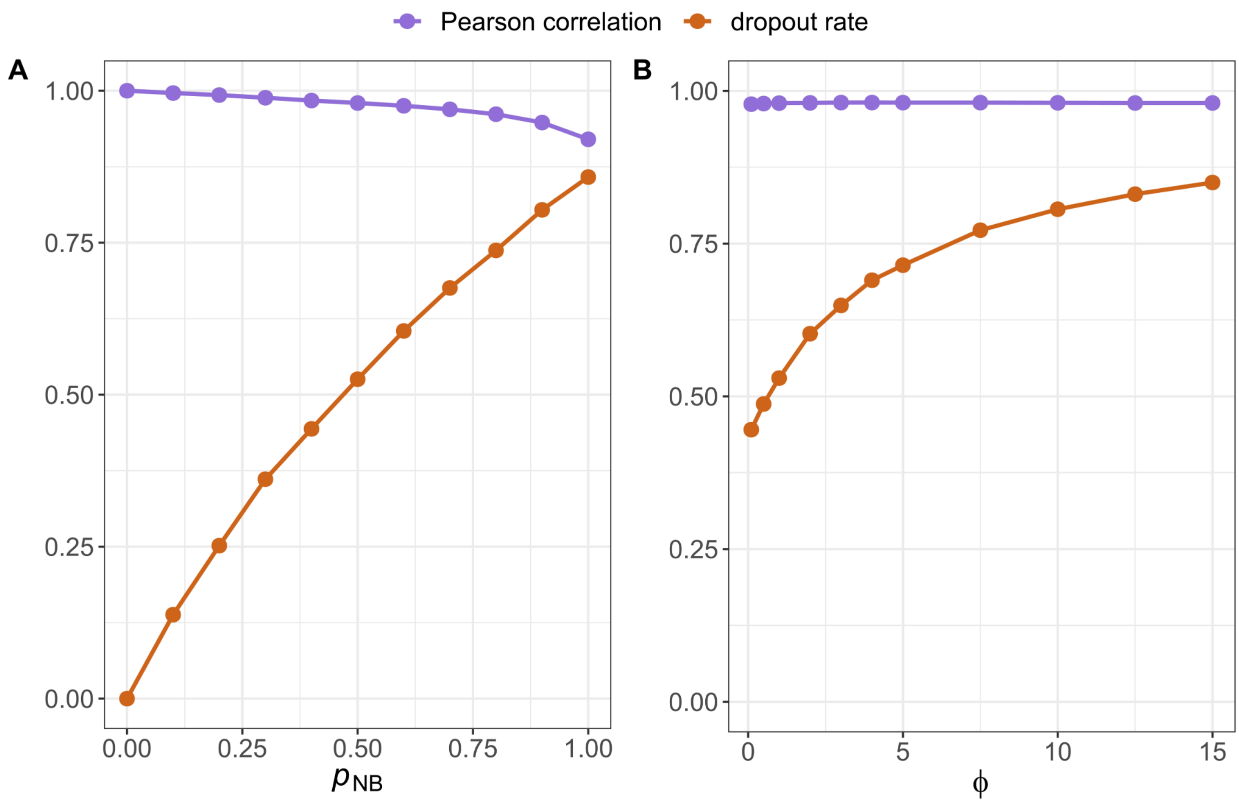

Figure S13. Pearson correlation coefficient between input (Y) and output (Y_{SC}) fold-change values, and overall dropout rate under **A**) different negative binomial probabilities p_{NB} for dispersion (ϕ) 1 and **B**) different ϕ for p_{NB} 0.5. Each estimation was done on a data set with 1000 genes, SNR_vov = 100, a GRN of sparsity 3 links per node, and 20000 cells. Each point is the average of 20 estimations.

Supplementary tables

Table S1. Inference methods available in GS2.

No.	Method	Option in GeneSPIDER	Perturbation design
**1**	Adaptive boosting	*adaboost*	✓
**2**	Bootstrap Aggregation (Bagging) and Random Forest	*bag*	✓
3	Classification And Regression Tree	*CART*	✓
4	Elastic Net	*elnet*	✓
**5**	Constrained least squares with Newton	*fcls*	✓
**6**	Gentle Adaptive Boosting	*gentleboost*	✓
**7**	Graphical lasso	*GLasso*	✓
**8**	Gaussian process regression for linear function	*gplin*	✓
**9**	LASSO based convex programming	*julius*	✓
**10**	Least Angle Regression	*lars*	✓
**11**	Lasso	*lasso*	✓
**12**	Lasso for generalized linear model with Poisson distribution	*lassoglmp*	✓
**13**	Logistic learner with lasso regularization	*lassolog*	✓
**14**	Adaptive Logistic Regression	*logitboost*	✓
**15**	Least Squares Cut Off	*lsco*	✓
**16**	Least Squares Cut Off with Normalization	*LSCON*	✓
**17**	Neural network model-based testing	*neunet*	✓
**18**	Neural network training and testing in cross-validation process	*neunetcv*	✓

**19**	Ridge regression with Cut Off	*ridgeco*	✓
**20**	Robust network inference	*RNI*	✓
**21**	Support Vector Machine classification	*svmc*	✓
**22**	Total Least Squares Cut Off	*tlsco*	✓
**23**	Z-score based inference	*Zscore*	✓
**24**	Mutual information with ARACNE	*aracne*	✗
**25**	Bayesian bootstrapping based on mutual information	*BC3NET*	✗
**26**	Context Likelihood of Relatedness	*CLR*	✗
**27**	Regression trees with Genie3	*GENIE3*	✗
**28**	Partial least squares	*PLSNET*	✗
**29**	Support Vector Machine regression	*svmr*	✗
**30**	Trustful Inference of Gene REgulation using Stability Selection	*TIGRESS*	✗

Table S2. Overview of GRN-based single-cell data simulators.

<table>
<tr><th>Tool</th><th>GeneSPIDER2</th><th>GRouNdGAN</th><th>SERGIO</th><th>BoolODE</th></tr>
<tr><td>Authors</td><td>This paper</td><td>(11)</td><td>(12)</td><td>(13)</td></tr>
<tr><td>Language</td><td>MATLAB</td><td colspan="3">Python</td></tr>
<tr><td>Data type</td><td>knockdown</td><td colspan="3">knockout</td></tr>
<tr><td>Input GRNs</td><td>Internal simulator, with controllable modularity or external user-defined</td><td colspan="3">External user-defined</td></tr>
<tr><td>Simulates counts</td><td>Internally</td><td colspan="3">By reference gene expression data</td></tr>
<tr><td>Allows to control data clusters</td><td>By tuning their number and distance between them</td><td>✗</td><td>By tuning their number</td><td>✗</td></tr>
<tr><td>Contains built-in GRN inference methods</td><td>✓</td><td>✗</td><td>✗</td><td>✓</td></tr>
<tr><td>Noise level</td><td>User-defined based on several SNR models and other parameters</td><td rowspan="2">Reference data-derived</td><td>User-defined based on several parameters and tuned to reference data</td><td>User-defined based on strength s and other parameters</td></tr>
<tr><td>Noise model</td><td>Additive Gaussian (see main text)
+
Probabilistic dropouts (16)</td><td>Multiplicative in ODE equation
+
Probabilistic outliers
+
Scalable library size
+
Probabilistic dropouts (logistic function)</td><td>Multiplicative in ODE equation
+
Probabilistic dropouts (17)</td></tr>
</table>

Table S3. Gold standard regulatory networks used for verification of real properties. Last access on 16th January 2024.

<table>
<tr><th>Organism</th><th>Database</th><th>Version info</th><th>Author</th><th>Link</th></tr>
<tr><td>E. coli</td><td>RegulonDB</td><td>11.2</td><td>(18)</td><td>https://regulondb.ccg.unam.mx/</td></tr>
<tr><td rowspan="4">H. sapiens</td><td>RegNetwork</td><td>Release year: 2015</td><td>(19)</td><td>https://regnetworkweb.org/</td></tr>
<tr><td>HTRI</td><td rowspan="2">v1.0</td><td rowspan="2">(20)</td><td>https://tflink.net/</td></tr>
<tr><td>TRED</td><td>https://tflink.net/</td></tr>
<tr><td rowspan="2">TRRUST</td><td rowspan="2">2</td><td rowspan="2">(21)</td><td rowspan="2">https://www.grnpedia.org/trrust/</td></tr>
<tr><td>M. musculus</td></tr>
<tr><td>S. aureus</td><td rowspan="2">DREAM5</td><td>1</td><td rowspan="2">(22)</td><td>https://www.synapse.org/#!Synapse:syn2787242</td></tr>
<tr><td>S. cerevisiae</td><td>1</td><td>https://www.synapse.org/#!Synapse:syn2787244</td></tr>
</table>

Supplementary references

1. Sloan,C.A., Chan,E.T., Davidson,J.M., Malladi,V.S., Strattan,J.S., Hitz,B.C., Gabdank,I., Narayanan,A.K., Ho,M., Lee,B.T., *et al.* (2016) ENCODE data at the ENCODE portal. *Nucleic Acids Res.*, **44**, D726–32.

2. Sunshine,S., Puschnik,A.S., Replogle,J.M., Laurie,M.T., Liu,J., Zha,B.S., Nuñez,J.K., Byrum,J.R., McMorrow,A.H., Frieman,M.B., *et al.* (2023) Systematic functional interrogation of SARS-CoV-2 host factors using Perturb-seq. *Nat. Commun.*, **14**, 6245.

3. Replogle,J.M., Saunders,R.A., Pogson,A.N., Hussmann,J.A., Lenail,A., Guna,A., Mascibroda,L., Wagner,E.J., Adelman,K., Lithwick-Yanai,G., *et al.* (2022) Mapping information-rich genotype-phenotype landscapes with genome-scale Perturb-seq. *Cell*, **185**, 2559–2575.

4. Hao,Y., Stuart,T., Kowalski,M.H., Choudhary,S., Hoffman,P., Hartman,A., Srivastava,A., Molla,G., Madad,S., Fernandez-Granda,C., *et al.* (2023) Dictionary learning for integrative, multimodal and scalable single-cell analysis. *Nat. Biotechnol.*, 10.1038/s41587-023-01767-y.

5. Morgan,D., Studham,M., Tjärnberg,A., Weishaupt,H., Swartling,F.J., Nordling,T.E.M. and Sonnhammer,E.L.L. (2020) Perturbation-based gene regulatory network inference to unravel oncogenic mechanisms. *Sci. Rep.*, **10**, 14149.

6. Huynh-Thu,V.A., Irrthum,A., Wehenkel,L. and Geurts,P. (2010) Inferring Regulatory Networks from Expression Data Using Tree-Based Methods. *PLoS One*, **5**, e12776.

7. Tjärnberg,A., Morgan,D.C., Studham,M., Nordling,T.E.M. and Sonnhammer,E.L.L. (2017) GeneSPIDER - gene regulatory network inference benchmarking with controlled network and data properties. *Mol. Biosyst.*, **13**, 1304–1312.

8. Czanner,G., Sarma,S.V., Ba,D., Eden,U.T., Wu,W., Eskandar,E., Lim,H.H., Temereanca,S., Suzuki,W.A. and Brown,E.N. (2015) Measuring the signal-to-noise ratio of a neuron. *Proc. Natl. Acad. Sci. U. S. A.*, **112**, 7141–7146.

9. Peidli,S., Green,T.D., Shen,C., Gross,T., Min,J., Garda,S., Yuan,B., Schumacher,L.J., Taylor-King,J.P., Marks,D.S., *et al.* (2024) scPerturb: harmonized single-cell perturbation data. *Nat. Methods*, **21**, 531–540.

10. Rizzo,M., Székely,G. and Others (2022) E-statistics: Multivariate inference via the energy of data. *Energy*, **1**, 11.

11. Zinati,Y., Takiddeen,A. and Emad,A. (2023) GRouNdGAN: GRN-guided simulation of single-cell RNA-seq data using causal generative adversarial networks. *bioRxiv*, 10.1101/2023.07.25.550225.

12. Dibaeinia,P. and Sinha,S. (2020) SERGIO: A Single-Cell Expression Simulator Guided by Gene Regulatory Networks. *Cell Syst*, **11**, 252–271.e11.

13. Pratapa,A., Jalihal,A.P., Law,J.N., Bharadwaj,A. and Murali,T.M. (2020) Benchmarking algorithms for gene regulatory network inference from single-cell transcriptomic data. *Nat. Methods*, **17**, 147–154.

14. Schaffter,T., Marbach,D. and Floreano,D. (2011) GeneNetWeaver: in silico benchmark generation and

performance profiling of network inference methods. *Bioinformatics*, **27**, 2263–2270.

15. Csardi,G., Nepusz,T. and Others (2006) The igraph software package for complex network research. *InterJournal, complex systems*, **1695**, 1–9.

16. Svensson,V. (2020) Droplet scRNA-seq is not zero-inflated. *Nat. Biotechnol.*, **38**, 147–150.

17. Chan,T.E., Stumpf,M.P.H. and Babtie,A.C. (2017) Gene Regulatory Network Inference from Single-Cell Data Using Multivariate Information Measures. *Cell Syst*, **5**, 251–267.e3.

18. Salgado,H., Gama-Castro,S., Lara,P., Mejia-Almonte,C., Alarcón-Carranza,G., López-Almazo,A.G., Betancourt-Figueroa,F., Peña-Loredo,P., Alquicira-Hernández,S., Ledezma-Tejeida,D., *et al.* (2024) RegulonDB v12.0: a comprehensive resource of transcriptional regulation in E. coli K-12. *Nucleic Acids Res.*, **52**, D255–D264.

19. Liu,Z.-P., Wu,C., Miao,H. and Wu,H. (2015) RegNetwork: an integrated database of transcriptional and post-transcriptional regulatory networks in human and mouse. *Database* , **2015**.

20. Liska,O., Bohár,B., Hidas,A., Korcsmáros,T., Papp,B., Fazekas,D. and Ari,E. (2022) TFLink: an integrated gateway to access transcription factor-target gene interactions for multiple species. *Database* , **2022**.

21. Han,H., Cho,J.-W., Lee,S., Yun,A., Kim,H., Bae,D., Yang,S., Kim,C.Y., Lee,M., Kim,E., *et al.* (2018) TRRUST v2: an expanded reference database of human and mouse transcriptional regulatory interactions. *Nucleic Acids Res.*, **46**, D380–D386.

22. Marbach,D., Costello,J.C., Küffner,R., Vega,N.M., Prill,R.J., Camacho,D.M., Allison,K.R., DREAM5 Consortium, Kellis,M., Collins,J.J., *et al.* (2012) Wisdom of crowds for robust gene network inference. *Nat. Methods*, **9**, 796–804.
